# Supplementary material for: Patterns of Intron Gain and Loss in Fungi
Source: PLoS Biol. 2004 Nov 30;2(12):e422. doi: 10.1371/journal.pbio.0020422 (PMC532390; doi:10.1371/journal.pbio.0020422)
Supplement: Table S1 — Also available at http://genes.mit.edu/NielsenEtAl/. (4.3 MB ZIP). [file pbio.0020422.st001.zip › NielsenEtAl/html/519.html]

AN6738.1.NCU04463.1.MG05095.1.FG05671.1


```
 CLUSTAL W (1.82) Multiple Sequence Alignments - Introns Inserted


Sequence 1: NCU04463.1	1441 aa
Sequence 2: MG05095.1	1415 aa
Sequence 3: FG05671.1	1402 aa
Sequence 4: AN6738.1	1411 aa
Alignment Length: 1506 aa
Number Identitical Residues: 425 aa
Alignment Score (without introns) 25442


MG05095.1 	-----MATQTPMREVPGAFLATPAIAARNDR----VSRQLFDTSRPSAGSRTSSSTALGV
NCU04463.1	-----MNPATPMRQVPGAFLNTPAAPRTDDARRYLDSLPEKNTARPVLGGFGSYNAGGAG
FG05671.1 	---MSFPQTTPVRPVPGAFLNTPAVTSRYQQGSDPVRRQLFPVSESGQSVSSLKSTAPTS
AN6738.1  	MSYPSLPPVTPQRPLPGAFFQTPAPG---------------NALNPPQSNSAQQTAQPPS
          	 :  ::   ** * :****: ***                  . ..  .     .:    

MG05095.1 	RMSSPRAGAMTSVTSGQGTDAAAP-------------------LPPLLKAAKSVKGVLQQ
NCU04463.1	VASGPVTGPVSGSVGGAGAGTVAAPRPVSQVPVPMVPAQARTDLPPVAKAARRITNTLLR
FG05671.1 	SASRGVTVTGSSVVRPSSTDGLLVP--------TVLPPLRSENVPPVIKAAKAINTCLQA
AN6738.1  	DAGQPVLPRLPPSIKPAPQN-----------------------LNTEERAARTINDTLTQ
          	  .       .        .                       : .  :**: :.  *  

MG05095.1 	DESYPDLDSVCRP1GATSDYDLQ-FADSPLAPFQRAQMYPIPDRIFETLNMSNLTTKLGL
NCU04463.1	DENYPDVDSYARA1GSSSDYDMYRTDDAPGAPFKKTHMYPIPDQVFERLNRGEVNTQLGL
FG05671.1 	DGRYPDLDSYCRQ1GASSDYDLE-STDSAMAPFHKTQMYPIPNQVFDHYNAGELQTLMGL
AN6738.1  	EARYPDLDSYLSQ1GFSSDYDIP--VSSTWAPFQKVRMYNIPDQIFDQYNLAQVSTSMGL
          	:  ***:**     * :****:    .:. ***::.:** **:::*:  * .:: * :**

MG05095.1 	FAEIGYAYACIDNALLMWNFSQPNPEIIGFEDQPHSIQTVALVPPKPGVFLSSITHILVV
NCU04463.1	FADINYAWAAIDSSLFLWDYTHPNPELIGYEDATTTITAVALVPPKPGVFVSNITHILVV
FG05671.1 	FAEINHAWVVIDNSLFLWDYTHPDPELIGFEDSPHTIHAVALVPPKPGIFVGSITHILVV
AN6738.1  	LAELNHAWVAIDNALYLWDYTHTNPQLVGFEDQPNSINAVKLAKPRPGVFLPSITHLLVI
          	:*::.:*:. **.:* :*::::.:*:::*:** . :* :* *. *:**:*: .***:**:

MG05095.1 	ATSQDLILLGIAASSSPNGLKTIELYSTKMALGTPSSHVSHIVGT-SAGRIFFGSSDETD
NCU04463.1	ATGTEIVLLGVSATPTPSGSKTVALYQTKMAVHRGSTDVSLIIGT-SNGRIFFGGYTDTD
FG05671.1 	ATSQEIVLLGVSATDTPSGTKSVNLYQTKMNLPLRGTDVRVITGS-SNGRIFFGGSNDID
AN6738.1  	STTAEVLLLGMKCEPTPGGSYQVTLYQTGMSTSVRGLDIHIIASSDATGRIFFAGSSDND
          	:*  :::***: .  :*.*   : **.* *     . .:  * .:.: *****..  : *

MG05095.1 	IYELCYQQEEKWFSGRCSRINHSQPGWSSVMPRLPASLDIWSPQKNEFLIDIKVDNSRNL
NCU04463.1	IYELLYQQEERWFSSRCAKVNHTHPGWTSVVPNLPVPMGFLGHRDQEHLVQLVVDDTRKL
FG05671.1 	INELYYQSEEKWFSNRCGKINHTNPGWSSVVT---LQGSFWSQKSPEHLVDIVIDDSRNL
AN6738.1  	VYELTYQQEEKWFQGRCSKINHTSSRISQLAP-----SLSFTQRPSEQVVQMVMDDSRRL
          	: ** **.**:**..**.::**: .  :.: .           :  * :::: :*::*.*

MG05095.1 	LYTLSNKSTIRTYHIETPDRLTCVITKEKNHCLRDVTHWN--HPSPLLTDAMAIVAISPI
NCU04463.1	LYSLSDKSTIRTYFMEPSGKLTKVIEKNKVSCLSDMTHMVDLNRNPLLNERVRIVSLSAI
FG05671.1 	VYTLSSRSTIRTYHMEAPDRLNKVIEKEKVHCLRDIAHMIS--QSRLLNDRVEIVAISPI
AN6738.1  	LYTLSSASTIRVFHLRADGSVALAITKPALDIYANIGHIIA--SNQALNSKVRIVSISPI
          	:*:**. ****.:.:.. . :  .* *       :: *      .  *.. : **::*.*

MG05095.1 	PATEASKLHLMALTSTGCRLFLSATSSASYMINTSSSLAPQSMQVQFVKFPPKDLN----
NCU04463.1	SAQEASKSHLMALTNTGCRLFLTATSAASYMLSASTSLAPQSMQVQYIKFPPTSEDQQVP
FG05671.1 	SKQEAAKLHLMALTNTGCRLFFSATSAASYLYGSSTNLAPQSMQVQFIKFPPGHRSP---
AN6738.1  	PAAEASRYHLMATTATGYRIYLSATGSYSWSPAPNGANAPTSMQAHYVKTPPFDSSAP--
          	.  **:: **** * ** *::::**.: *:   ..   ** ***.:::* **   .    

MG05095.1 	-RDRRNTG--YDSSGDSLTDLQSRSLEVSELGFRFPPGYFIDFVRKPGTPDTTTIFASAP
NCU04463.1	LRNEINTAVAYAPAPAAAQDQMSGLLSESCLGERFPPGYFFDAVR-PKESRTDLLFVSAP
FG05671.1 	----------YAGS-ETTIDLDSNSLTWSRQGARFPPGYFLDFVRKESNPNEDSLFISGP
AN6738.1  	----AYQGQSRFQPSLAATRVPIHTLDPTSSSVRYPPGYFFCFTCQDSTQKSDTLFISAP
          	       . :   .  :        *  :  . *:*****:  . .        :* *.*

MG05095.1 	ETGRIKAIGA-ASALKFYEHANWIDIGPKTVALDVGLINKPFAAAKQPLGFGNELAVQFD
NCU04463.1	DVGRLKQS----AVLRYVEQGTFIPLGDGSRVLSIGLTGNPFAAAKQPLGFGNELAVQFD
FG05671.1 	ETGRLKNTLP-TSPLKYHESGIWIDIG--SRAEAVGIITKPFAAASQPLGFGNELAVQFD
AN6738.1  	DSGRVARSQENVIPGKAAETGIWLSLG--SRMEDIGLSSPTTPASATPSGFGNELAVQFE
          	: **:     .    :  * . :: :*  :    :*:   . .*:  * **********:

MG05095.1 	EDPCEFAILTNSGIYSVRRRRFVDIFANTMR--VTVGEESLDKDIRKFIQLYGRVETVSA
NCU04463.1	HTPSEFAVLTNTGIHIVRRRRFVDIFANAIR--VASGDEDLDSKVKTFTAIYGRVEMVAA
FG05671.1 	DAPSEFAVLTNTGVHIIRRRRFVDIFASAIR--GAVGDEGLELVCRRFINTYGRVETVTT
AN6738.1  	NPSAEIAILTNTGIHVFRRRRLVDIFAALVRNGGSGGEEGLEGEIKNFIRTYGRSETLAT
          	. ..*:*:***:*:: .****:*****  :*.. : *:*.*:   : *   *** * :::

MG05095.1 	ALAVACGQGTDLRTGT--SRVMDQGTADRAKSVFVNFGGQPLLAE---SDGAISPE-SVK
NCU04463.1	ALAVACGQGGDAR------RAVDPLTEDRARSIFINYGGQPKLAD---ADVLQAPEDNVQ
FG05671.1 	ALAVACGHGGDSRPGA--ARAIDQATEDRARSVFVDFGGQPTMAE---TDSASLTTESVN
AN6738.1  	ALAVACGQGVEVSTDSRLSKINDPDVLEFARKVFIDYGGRPTLNENAVADNSTPAIDTVV
          	*******:* :  ..:  ::  *  . : *:.:*:::**:* : :.: :*    . ..* 

MG05095.1 	LSSRHDALALYLTRLIRSLWKAKVITARVDANAVGGVVVSSTISTAKLTIVQENIERLRN
NCU04463.1	LSARHDALVLYLARLARTLWKSKVIEIATDS-ASGGFTVGSAIPISKLMTVQESVQRLRR
FG05671.1 	LSSRHDALALYLSRLIRQLWKSAVITPGVSP--TGGITIGSTIPLTKLSTVQEALERLRR
AN6738.1  	PSPRHAGIALYISRLLRSVWKKEVAVVGGGP--GGAQTISPSFPRAKLLAIQHDLSALQE
          	 *.** .:.**::** * :**  *     ..   *. .:..::. :**  :*. :. *:.

MG05095.1 	FLNTNKGIIQGLSGPADLQRVSSRQEEVALQAEHQALHALQKLMESISEGISFVLMLFDE
NCU04463.1	FLEENKSLIQGLAGPSD--RVSSRQEEIALQLEHQSLHALRQLMDSVSEGISFVLMLFDE
FG05671.1 	FLDSNRGLIQGLSGPSDLQHVSSRQEEIALQAEHQALHALQKLMESISEGISFVMMLFDE
AN6738.1  	FFKTNKSFIEGLSGPEALARVSTKQEELELQAEHRALHSLVQLVSHTIEGISFVTVLFDE
          	*:. *:.:*:**:**    :**::***: ** **::**:* :*:.   ****** :****

MG05095.1 	RVTDIFLRLESNTRDELKELTYESLFSQPAGKDLAKLLVKAIVNRNIESGSNVETVADAL
NCU04463.1	RVTDIFMRLDDSSKQQLQNLTYEALFSQDSGKELAKVLVKAIVNRNIANGANVETVADAL
FG05671.1 	RVADIYTRLDDTARQQLKDLTYEKLFSQTDGKDLAKLLVKAIVNRNIESGSNVETVADAL
AN6738.1  	KVEEIVALLPEESKQRFLKLTYEELFSTTKGHDIAKELVKGIVNRNIAKGANVETVADAL
          	:* :*   * . :::.: .**** ***   *:::** ***.****** .*:*********

MG05095.1 	RRRCGSFCSPDDVIIFKAQEQLKRASEQTLNHSSQRTLLSESLRLFERVAGNLTYANLRS
NCU04463.1	RRRCGTFCSPDDVVIFKAQEQLQRASESSGNPNLMRTLLAESLRLFSQVAGSLTMTNLRN
FG05671.1 	RRRCGSFCSPDDVVTFKAQEQLKRASDQPLQTNQSRSLLHESLRLFEKVAGSLTFANLQT
AN6738.1  	RRRCGSFCSSEDVVIFKAQELLKRATEAGFNSELGRNLLNESLHLFQQVSENLPMDYLVP
          	*****:***.:**: ***** *:**::   : .  *.** ***:**.:*: .*.   *  

MG05095.1 	AAEQYTDLKYYAGAIRLCLVVAKEKDRGNAALAWINENKPAN~DPREKIHTERQRCYDLV
NCU04463.1	AVEQYINLKYYAGAIQLCLIVAREKDRGNAALAWFNEGKPAN~DPREKAFKDRQSCYSLI
FG05671.1 	AVQQYIDLKYYAGAIQLCLVVAREKDRGNTALSWVNDGKPSS~DPRANAFNDRKRCYDMI
AN6738.1  	AVESYISNQFFAGAIQLALNVAGRSDKANLGLGWMMDGQPSS0------------CYDLI
          	*.:.* . :::****:*.* ** ..*:.* .*.*. :.:*:.             **.::

MG05095.1 	HAVLERLDAESSKEPELVDGKPSLAATKRMEAYSVVNDSDDEVFHFGLYAWYVEQNWTDR
NCU04463.1	HEVLDRLEIESASEPDVVDGRPTLAATKRAEAYSVVNSSDDEVFHLDLYDWYIEKGWTDR
FG05671.1 	HDVLSHLDAASSSEPEMVDGRLTLIATKRLEAYKVVNESDDEVFHFDLYEWYIQQGWTDR
AN6738.1  	FKVVLAVDNLAAQDPGVVDGQLTTIAKRKNEAYGIIAVSEDQVFLTSLYDWYLEQGWSDR
          	. *:  ::  ::.:* :***: :  *.:: *** ::  *:*:**  .** **:::.*:**

MG05095.1 	ILAIDSPHVITFLKRLAGSDVRHAELLCRFYTHRGQFYDAAQVQAELANSEFDIGIKDRI
NCU04463.1	MLAIDSPHVVTYLQRLAAVDAQHADLLCRFYTQRSRFFEAAQVQAELAKSDFPLGIKERI
FG05671.1 	ILAIDSNHVVTFLQRLAGTNIEHADLLCRFYTNRSRFFDAAEVQAELANSDFPISIKDRI
AN6738.1  	LLRSESPYVVEYLKRKSTDDISHADLLWRYYTQSERFFEAAQVQLELAQSSFVLPLSRRI
          	:*  :* :*: :*:* :  :  **:** *:**:  :*::**:** ***:*.* : :. **

MG05095.1 	TLLSRAKGNASVMGAGGNRQQQQLLSHEISELLEIAHIQDDLLERLKADPRIADERKMEI
NCU04463.1	TLLSRAKANASVTTVGVSRQQQQLLNHEVTELLEIAHIQDDLLERLRADPRIVPEKLPDI
FG05671.1 	RLLSLAKANANVSTTGISRQQQQMLNHSVTELLEIAHIQDDLLERLRADDRIDPERAIEI
AN6738.1  	EYLGQARANASTFTQDVGRQARQRLLQQVSNLIDVANVQDDLLQRLKDDTRLDPDAKARA
          	  *. *:.**..   . .** :* * :.:::*:::*::*****:**: * *:  :     

MG05095.1 	EKVLDGQIQGLTE0LFNTYADPACYYDLCLLVYHAADYHNTRLIADTWKNLIDATHREIL
NCU04463.1	EKVLDGPVQGLST0LFNDFADQAGYYDLCLLIYNAADYQNPHTIKETWENLINQIHFETE
FG05671.1 	EDALKGKIQGLSE0LFNDYADQAGYYDLCLLIYHVADYRNHMTISGTWSNLIQQTHDEVM
AN6738.1  	VEEVDGPIMEVST0MFNQYADVASYYDICLQIFYIADYRNPADVRATWQHLLQTLHDEAV
          	 . :.* :  ::  :** :** * ***:** ::  ***:*   :  **.:*::  * *  

MG05095.1 	ERHEAYNEYENSGGRLPPGVTVPPSPPPLPYEAVSMQIQNIAHRTSLDTMIFPIDTLLPI
NCU04463.1	QKQEVWRLIE-AGEALPEGVEALEVPPPMPYEAVVSQIQSIAHRTSLDSLIFPVDTLLPM
FG05671.1 	SR------LEMSEPGMPS--------PPLPYECVTSKIQNIAHRTSLDSFIFPIQDLLPE
AN6738.1  	ARG---------------------SAGPQPYEAVIDKLRSLGARLRMSDAVFPIPILLPM
          	 :                       . * ***.*  :::.:. *  :.  :**:  *** 

MG05095.1 	VCEYAVAESQDESIGADNSWPVLLFLQLQVSQSVIVKILERIFDAQEAPFTGRKKRIVAE
NCU04463.1	VCAYAINNHQDASIGADPCWPVAAFLQLGVPHALIARVLENMFDAQEAPFVGKHRKLVVQ
FG05671.1 	LCRYAVAYQQDATIGADPTWPVQLFLTLGVSHDMIVRVLEGVFDSQDYGFSGSVRNRMIE
AN6738.1  	LERYALEHQRNVGP---PTWVIDTFFDLGVPHETIYSVLESMYYIDEAPFHGSNRKIIAR
          	:  **:   ::        * :  *: * *.:  *  :** ::  ::  * *  :. : .

MG05095.1 	WINAAVESWLREVERQG--SGKQ-DGALSSWVFELLGRVDEEMLQLARSVRDSSVKQSAE
NCU04463.1	WINCVVDSWIREIEHRGSASGKNGDGAITTWVAELLTRAEEALVAIAAGTRDPAQAASIT
FG05671.1 	LIVYVVNDWVAEARRRG---GAGKGGAIGPSVADLLKRCDAALPPPGHGN--NAGGADLA
AN6738.1  	DLLYTIEHWFHDTVRLG--GVVFGSDLIAERIEEMLTLLQQSGISAEQMAVAHELRARIN
          	 :  .:: *. :  : *  .    .. :   : ::*   :                    

MG05095.1 	KVGFR~ARQLRN----------------TVDRAMRSTNVTGSMRYL--------------
NCU04463.1	DTRRD~TRELKR----------------KVE-MLSQAEAQGSLLFSTR------------
FG05671.1 	DVRRV~LKGLKR----------------EVD-GLSQRVPTGSLRFA--------------
AN6738.1  	NILRY2KAAAYSPDIRLLLPALAMYVSSNIDQWTKLVDVPKPIYFLFLPFSDYMSTMKTG
          	.           ..     .: :   :: ::          .: :   . :.  :: .:.

MG05095.1 	--~--------
NCU04463.1	--~--------
FG05671.1 	--~--------
AN6738.1  	RV0FAMPQAKG
          	    : ..:..
```
